# *Cutibacterium acnes* Culture Isolation Following Total Hip and Total Knee Arthroplasty

**DOI:** 10.3390/antibiotics15020165

**Published:** 2026-02-04

**Authors:** Benjamin Levy, Alton Daley, Tracy Borsinger, Paul Werth, Wayne Moschetti

**Affiliations:** 1 Dartmouth Geisel School of Medicine, 1 Rope Ferry Rd, Hanover, NH 03755, USA; benjamin.a.levy.med@dartmouth.edu; 2 Department of Orthopaedic Surgery, Dartmouth Hitchcock Medical Center, 1 Medical Center Drive, Lebanon, NH 03756, USA; alton.b.daley@hitchcock.org (A.D.); tracy.borsinger@orthovirginia.com (T.B.); paul.werth@orthovirgina.com (P.W.)

**Keywords:** prosthetic joint infection, revision total knee arthroplasty, revision total hip arthroplasty

## Abstract

Introduction: *Cutibacterium acnes*, a component of normal skin flora and a common commensal Gram-positive bacterium, presents a diagnostic challenge for arthroplasty surgeons. While *Cutibacterium acnes (C. acnes)* as a source of infection has been well characterized in shoulder surgery, its presentation and clinical significance in total hip (THA) and total knee arthroplasty (TKA) remain less understood. Methods: A retrospective chart review identified patients with *C. acnes* culture positivity following THA or TKA. Demographics, laboratory values, and microbiologic data were collected. Statistical comparisons were performed using *t*-tests and chi-squared analysis. One-year outcomes were evaluated using the Musculoskeletal Infection Society Outcome Reporting Tool (MSIS ORT) criteria among patients undergoing further surgical intervention. Results: Twenty-nine patients with *C. acnes*-positive cultures were identified (21 THA, 8 TKA); 15 (52%) were polymicrobial. Ten THA patients (47.6%) and seven TKA patients (87.5%) met MSIS criteria for infection at the time of presentation. Mean time to culture positivity was similar between THA (6.8 days) and TKA (7.4 days; *p* = 0.57). Sonicated cultures were positive in 24% of THA and 12.5% of TKA cases. Mean ESR was 36.4 mm/h for THA and 51.5 mm/h for TKA (*p* = 0.21); mean C-reactive protein (CRP) was 35.2 and 36.8 mg/dL, respectively (*p* = 0.95). Mean synovial cell counts were 27,055 for THA and 22,194 for TKA, with polymorphonuclear cells (PMN) percentages of 68% and 73.9% (*p* = 0.72, 0.70). Monomicrobial infections demonstrated a mean cell count of 24,143 with 58.9% PMNs, compared to 25,903 and 78.8% in polymicrobial cases. At one year, 72% of patients undergoing subsequent surgery achieved successful outcomes. Higher ASA classification was the only significant predictor of failure (mean 3.0 vs. 2.75). Conclusions: *C. acnes*-associated THA and TKA infections often present with delayed culture growth, mild inflammatory markers, and frequent polymicrobial involvement. At one-year, patients with available follow-up who undergo surgical management experience favorable outcomes, with 72% achieving MSIS ORT success.

## 1. Introduction

Infection following total hip arthroplasty (THA) and total knee arthroplasty (TKA) remains an important area of study, given the significant costs related to these procedures as well as the morbidity associated with clinical management [1,2,3]. While studied more extensively in orthopedics as a pathogen following shoulder surgery [4,5], *C. acnes* following THA and TKA remains poorly understood with respect to the unique characteristics of the organism and variable clinical presentations. *C. acnes* species, therefore, poses an interesting diagnostic challenge for orthopedic arthroplasty surgeons.

*C. acnes* species, formerly known as *Propionibacterium* species, are commensal Gram-positive skin bacterium which can cause implant-associated infections. They are ubiquitous organisms present in skin flora and are generally found in sebaceous glands. They are notably slow growing, facultatively anaerobic, and non-spore forming. Techniques such as prolonged agar cultures for 14 days, as well as tissue sonication, have increased the detection of these bacteria [6]. Although once thought to be a cutaneous bacterium of little pathologic significance, there has been increasing evidence for its role in both native as well as implant-associated infections [7,8].

Notable prior work has evaluated *C. acnes* in shoulder arthroplasty [9,10,11,12,13,14] as well as infections following anterior cruciate ligament reconstruction [15], shoulder arthroscopy [16,17,18], and clavicle non-unions [19]. However less has been reported about *C. acnes* in total joint arthroplasty. Elkins et al. assessed anterior and lateral thigh skin colonization of *C. acnes* prior to total hip arthroplasty and found that 14 of the 101 (14%) patients had a positive culture, with most localized to the anterior hip (65%) [20]. Prior work by Nodzo et al. compared infection characteristics for patients undergoing revision TKA for infection due to *C. acnes* versus methicillin sensitive *Staphylococcal aureus* [21]. Although a small study of 16 patients with *P. acnes*, they did demonstrate that median ESR was significantly higher in the MSSA group (56.0 mm/h; interquartile range [IQR], 44.3–72.9 vs. 23.0 mm/h; IQR, 18.5–52.0, respectively, *p* = 0.03). Similar findings were noted for CRP; however, synovial and serum WBC count did not demonstrate any differences between the two groups. This work also noted a mean time to culture of 8.3 ± 2.0 days in the *C. acnes* group compared to 1.8 ± 0.8 days in the MSSA cohort.

Given the potential for varied and perhaps less overt clinical symptoms in patients with *C. acnes* prosthetic joint infection (PJI), this study sought to evaluate TKA and THA *C. acnes* infections with respect to presenting characteristics, objective clinical workup, treatments, and outcomes of patients undergoing surgical intervention at the 1-year mark.

## 2. Results

Retrospective review identified a total of 29 patients with positive *C. acnes* cultures during the study period, including 21 total hip arthroplasties (THAs) and 8 total knee arthroplasties (TKAs). The mean age was 65.8 years for THA patients and 64.7 years for TKA patients (*p* = 0.82). Preoperatively, 10 of 21 THA patients (47.6%) met MSIS criteria for prosthetic joint infection, compared with 7 of 8 TKA patients (87.5%) (*p* = 0.13). Seven of eight patients with TKA infection (87.5%) were female. There were no statistically significant differences between THA and TKA cohorts with respect to age, sex, ASA class, BMI, or history of prior revision surgery (Table 1).

Microbiologic evaluation demonstrated polymicrobial infection in 47.6% of THA cases and 62.5% of TKA cases, with *C. acnes* identified as one of the isolated organisms (Table 2). Mean time to culture positivity was 6.8 days for THA and 7.4 days for TKA (*p* = 0.57), reinforcing the importance of prolonged incubation. The mean number of positive cultures per case was 2.05 for THA and 1.75 for TKA (*p* = 0.66). Sonication cultures were positive for *C. acnes* in 23.8% of THA cases and 12.5% of TKA cases (*p* = 0.24).

Inflammatory markers demonstrated substantial variability. Mean ESR was 36.4 mm/h for THA and 51.5 mm/h for TKA (*p* = 0.21), while mean CRP was 35.2 mg/dL and 36.8 mg/dL, respectively (*p* = 0.95). Synovial fluid analysis demonstrated a mean cell count of 27,055 cells/µL with 68% PMNs for THA and 22,194 cells/µL with 73.9% PMNs for TKA (*p* = 0.72 and *p* = 0.70). Monomicrobial infections demonstrated lower mean PMN percentages compared with polymicrobial infections (58.9% vs. 78.8%) (Table 2).

### Management Strategies and Outcomes

**Figure 1:**
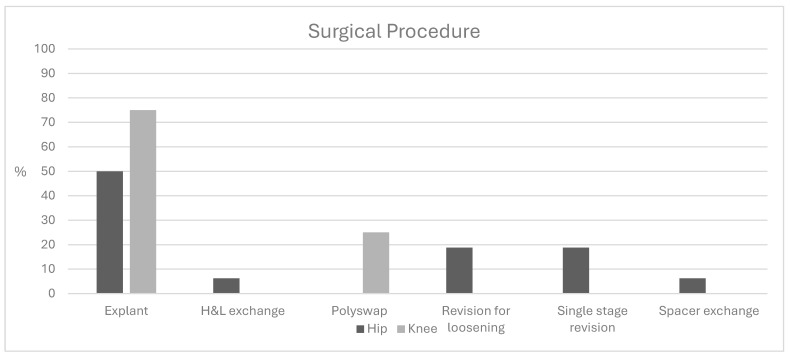
Subsequent surgical procedures performed following positive *C. acnes* culture.

Overall, 24 of 29 patients (82.7%) underwent surgical intervention following identification of a positive *C. acnes* culture (Figure 1). Surgical strategies included component explant with planned staged reconstruction (n = 14), revision for aseptic loosening (n = 3), polyethylene exchange (n = 2), spacer exchange (n = 1), single-stage revision (n = 3), and head and liner exchange (n = 1). Explantation was more common in TKA patients (75%) compared with THA patients (38.1%), though this difference was not statistically significant.

For patients with positive *C. acnes* THA cultures, the approach for the primary THA procedure was also further investigated (Table 3). Of the 21 patients in our study, 10 underwent prior THA via an anterior approach, and 8 underwent THA via a posterior approach (*p* = 0.92). Five of the ten (50%) anterior approach patients had monomicrobial infections, and similarly, four of the eight (50%) posterior approach patients had monomicrobial infections.

Five patients (17.3%) were managed non-operatively with antibiotics alone. Indications for conservative management included low clinical suspicion for active infection despite culture positivity (suspected contaminant), patient comorbidity burden precluding surgical intervention, or patient preference. Antibiotic regimens were organism-directed and guided by infectious disease consultation; however, heterogeneity in agent selection and duration precluded comparative analysis.

At one-year follow-up, outcomes for these patients were categorized using the MSIS Outcome Reporting Tool (ORT, Table 4), with the majority classified as Tier 2 (infection control with suppressive antibiotic therapy). No patients in the non-operative cohort progressed to reoperation within one year.

Among the 22 patients who underwent surgical intervention and had a one-year follow-up, outcomes were assessed using the MSIS ORT. Sixteen patients (72%) achieved successful outcomes (Tiers 1 or 2). When stratified by surgical strategy, patients undergoing single-stage revision or component explant followed by staged reconstruction demonstrated higher rates of Tier 1 or Tier 2 outcomes compared with those requiring spacer exchange or repeat reoperation, although the study was underpowered for formal statistical comparison.

## 3. Discussion

Detection and management of *C. acnes* prosthetic joint infection can pose a diagnostic challenge for orthopedic arthroplasty surgeons, given more subtle clinical as well as laboratory changes compared to more virulent microbial species, particularly in monomicrobial communities [22,23,24]. Our study showed that half of the THA and half of the TKA patients with *C. acnes* presented with polymicrobial infections, with the most common concomitant bacteria being coagulase-negative Staphylococcus. This is consistent with prior work demonstrating *C. acnes* as an overall rare monomicrobial pathogen that is not infrequently found in conjunction with other microorganisms [25,26]. We compared laboratory values for monomicrobial and polymicrobial *C. acnes* infections in THA and TKA patients and did not find statistically significant differences. However, the average CRP of the monomicrobial group was lower, with an average of 26.5 vs. 44.2 mg/dL for the polymicrobial group, regardless of the joint, supporting the potentially indolent nature of the bacteria. There was no significant difference between the frequency of polymicrobial PJI in THA and TKA patients, with 5 out of 8 TKA patients and 10 out of 21 THA patients having polymicrobial *C. acnes* infections. Further, our work showed a delay in time to culture-positivity of approximately 1-week, necessitating the need for extended cultures to assist in diagnosis (mean of 6.5 days for THA and 7.39 days for TKA).

White blood cell count, erythrocyte sedimentation rate (ESR), C-reactive protein (CRP), and synovial fluid polymorphonuclear leukocyte (PMN) percentage were, on average, elevated in both THA and TKA patients with *C. acnes* culture positivity. Mean ESR and CRP values exceeded MSIS diagnostic thresholds (>30 mm/h and >10 mg/L, respectively) [27]. However, when considered in isolation, only 47% of THA patients met MSIS criteria for prosthetic joint infection preoperatively, compared with 87% of TKA patients. This finding highlights a potential diagnostic challenge in THA, wherein *C. acnes* culture positivity may represent true infection—particularly in cases of monomicrobial growth—yet may also reflect contamination in the absence of corroborating clinical and laboratory findings. Ultimately, we believe this finding outlines the concept that conventional inflammatory marker values may be less reliable in the setting of *C. acnes* infection.

Prior studies have shown *C. acnes* infections to be more common in males than females who have had shoulder surgery [28]. This is consistent with the fact that *C. acnes* is present in areas with greater concentrations of sebaceous glands, and men have a greater concentration of sebaceous glands than women [29]. Our data does not show a statistically significant difference in men compared to women with regard to TKA or THA infections with *C. acnes*. This discrepancy could be related to the relative abundance of *C. acnes* around the axilla and shoulder region compared with the hip and knee. Using this theory, other studies have compared the difference between anterior and posterior approaches for THA and the relative rates of *C. acnes* infections. The theory was that perhaps there would be a higher rate of *C. acnes* culture positivity with the anterior approach, given the larger concentration of sebaceous glands in this region. One study found increased rates of *Cutibacterium avidum* [30] in patients with an anterior compared to a posterior THA. Our study did not find a statistically significant difference in the rates of *C. acnes* infections based on the approach of the initial arthroplasty procedure. Further studies have shown that the direct anterior approach to primary THA does not increase the risk of periprosthetic joint infection compared to the direct lateral approach. Additionally, there were not significant differences in infecting organisms between anterior and direct lateral approach groups [31]. Interestingly, of the 29 arthroplasty patients with *C. acnes* infections in this study, 21 were from THAs, with just 8 from TKAs. This is consistent with the study by Elkins et al., mentioned in the introduction, which showed that the anterior thigh has a high colonization rate of *C. acnes* [20].

This study has several limitations as a retrospective analysis of a relatively uncommon organism culture in THA and TKA. As mentioned, *C. acnes* are ubiquitous organisms present in skin flora, and some have suggested that isolation of the bacteria may represent a contaminant [32,33]. The present study sought to evaluate all patients with a positive culture, though we do acknowledge that of the 21 patients with THA infections, 10 preoperatively met MSIS criteria for PJI (47.6%), while 7 out of 8 patients with TKA infections preoperatively met current MSIS criteria for infection (87.5%) (*p* = 0.13). This finding may be explained by the less virulent nature of *C. acnes* to elicit common warning signs for PJI, such as significant erythema, effusion, or sinus tract, as well as the difficulty with culture of the bacteria. However, it is also possible that the *C. acnes* represents a contaminant or a non-pathologic organism, particularly in polymicrobial infections.

The purpose of this work was not to decipher such a difference but rather to report laboratory and other characteristics of those with a positive *C. acnes* prosthetic culture. Another limitation of our study is that it utilized conventional culture, which may have missed some positive identification of *C. acnes* possible with other methods (e.g., next-generation sequencing); however, we suspect the use of traditional cultures is more generalizable, given the feasibility of this method. Further, additional cases of *C. acnes* may be missed by cultures not being held for an extended time (14 days in our institution). As this study found, the average time to culture positivity was approximately one week (mean of 6.5 days with a range of 3–11 for THA and 7.3 days with a range of 5–12 for TKA). Lastly, there may have been other patient or clinical characteristics that influence the laboratory values and outcomes evaluated.

Lastly, given the small sample size observed in our study, despite rigorous statistical analysis, it should be noted that reported *p*-values are likely underpowered, increasing the possibility of type I and type II errors.

## 4. Methods

Retrospective chart review was performed to identify patients with *C. acnes*-positive cultures involving a total hip arthroplasty (THA) or total knee arthroplasty (TKA) treated at our tertiary academic referral center. Using the electronic medical record, all intraoperative or aspirate cultures positive for *Propionibacterium* or *C. acnes* between April 2014 and January 2021 were identified. Cases involving native joints or prosthetic joints other than THA or TKA were excluded (Table S1).

A *C. acnes*-positive case was defined as the presence of at least one positive culture obtained from synovial fluid aspiration, intraoperative periprosthetic tissue specimens, or sonication fluid in the setting of a THA or TKA. In accordance with institutional practice patterns, multiple intraoperative tissue specimens were routinely obtained when operative intervention was performed. Tissue and synovial fluid specimens were cultured using standard solid and liquid media under both aerobic and anaerobic conditions. Anaerobic cultures were specifically maintained to optimize recovery of slow-growing, low-virulence organisms such as *C. acnes* species. Culture plates and broth media were examined daily during the incubation period by our clinical microbiology laboratory. Notably, next-generation sequencing was not available as a diagnostic aid, limiting microbiological sensitivity.

Per institutional protocol for evaluation of suspected periprosthetic joint infection, cultures were incubated for a minimum of 14 days to enhance detection of slow-growing organisms (extended cultures). A single culture set was incubated for seven days due to laboratory workflow constraints at the time of processing; this deviation was recorded but not excluded, given the retrospective nature of the study. Identification of *C. acnes* species was performed using standard laboratory techniques in place during the study period.

For patients undergoing revision surgery with component removal, implant sonication was performed according to institutional standards. Retrieved components were placed in sterile containers, transported to the microbiology laboratory, and sonicated to dislodge adherent biofilm. Sonication fluid was subsequently cultured under aerobic and anaerobic conditions using the same prolonged incubation protocol. No predefined quantitative colony-forming unit (CFU) threshold was applied; any growth of *C. acnes* from sonication fluid was considered positive, consistent with contemporary institutional practice and the recognized low bacterial burden associated with indolent *C. acnes* infections. Low-level growth was interpreted in conjunction with clinical presentation, inflammatory markers, and MSIS criteria to distinguish likely contamination from clinically meaningful infection.

Cases with ≥2 concordant positive intraoperative tissue cultures were considered microbiologically consistent with infection. Cases with a single positive culture among multiple samples were retained for analysis, given the known low virulence, propensity for delayed growth, and established clinical relevance of *C. acnes* species in prosthetic joint infection. These cases were adjudicated using the 2018 Musculoskeletal Infection Society (MSIS) diagnostic criteria and integrated clinical, laboratory, and operative findings.

Using these criteria, 29 total cases were identified for further analysis. This study was conducted as a retrospective review of de-identified data. No patient names, medical record numbers, or other identifiable health information were collected, stored, or reported. As such, this project did not meet the definition of human subjects research under 45 CFR 46.102(f) and therefore did not require Institutional Review Board (IRB) approval.

Patient demographics, including sex, age, and body mass index (BMI), were collected. American Society of Anesthesiology (ASA) scores were used to assess overall medical comorbidity. Preoperative laboratory values included erythrocyte sedimentation rate (ESR), C-reactive protein (CRP), white blood cell (WBC) count, percentage of polymorphonuclear leukocytes (% PMNs), and alpha-defensin testing when available. Prior surgical interventions on the affected joint were recorded.

Using the 2018 Musculoskeletal Infection Society (MSIS) criteria for prosthetic joint infection, each patient was dichotomized as infected (MSIS score ≥ 6) or not definitively infected (MSIS score ≤ 5). One-year outcomes for patients who underwent surgical intervention and had documented clinical follow-up (n = 22) were categorized using the Musculoskeletal Infection Society Outcome Reporting Tool (MSIS ORT; Table 4). A single physician reviewer (TMB) retrospectively evaluated all charts and assigned patients to either “success” (Tier 1 or Tier 2) or “failure due to PJI or failure due to a secondary cause” (all remaining tiers).

### Statistical Analysis

Analysis of patient demographics, laboratory values, microbiologic findings, and surgical characteristics was performed for the overall cohort and to compare THA and TKA subgroups. Student’s *t*-tests and chi-squared analyses were used to compare baseline demographic and laboratory characteristics. Additional analyses evaluated one-year outcomes using the MSIS ORT among patients who underwent surgical intervention, with comparisons between “success” (Tiers 1–2) and “failure” (Tiers 3A–4B).

## 5. Conclusions

This study contributes to the current literature on *Cutibacterium acnes* culture positivity in TKA and THA patients. With longer culture times becoming more standard, *C. acnes* infections will continue to present themselves as a factor in PJI. This research illuminates some of the characteristics of monomicrobial and polymicrobial *C. acnes* infections in the hip and knee and demonstrates that *C. acnes* must be considered as a pathogen when suspecting a PJI in the hip and knee. We also acknowledge the exploratory nature of our findings and the potential for underpowering, given the small sample size. Further research is needed to identify just how prominent *C. acnes* infections in THA and TKA are and to identify the ideal treatments to optimize patient outcomes.

## Figures and Tables

**Table 1:** Patient demographic and baseline characteristics with comparison between THA and TKA.

Demographic	THA	TKA	*p*-Value
N	21	8	
Male Sex (%)	10 (47.6)	1 (12.5)	0.19
Age (mean (SD))	65.8 (9.32)	64.7 (12.0)	0.82
BMI (mean (SD))	34.6 (6.7)	31.2 (11.3)	0.47
ASA (mean (SD))	2.79 (0.43)	2.62 (0.52)	0.44
Prior revision (%)			0.23
No	17 (81.0)	5 (62.5)	
Unknown	0 (0.0)	1 (12.5)	
Yes	4 (19.0)	2 (25.0)	
Met MSIS Criteria (%)	10 (47.6)	7 (87.5)	0.13
Total Patients	21	8	

BMI = body mass index; ASA = American Society of Anesthesiology; MSIS = Musculoskeletal Infection Society.

**Table 2:** Laboratory data with comparison between THA and TKA.

Laboratory Data	THA	TKA	*p*-Value
Polymicrobial (%)	10 (47.6)	5 (62.5)	0.68
Time to culture positivity (mean (SD))	6.75 (2.5)	7.39 (2.6)	0.57
Number of positive cultures (mean (SD))	2.05 (1.6)	1.75 (1.5)	0.66
WBC count (mean (SD))	27,055 (3.9922)	22,195 (2.9172)	0.77
PMN % (mean (SD))	67.92 (36.5)	73.88 (29.3)	0.70
Sonicated tissues positive for *C. acnes* (%)			0.24
No	16 (76.2)	7 (87.5)	
Yes	5 (23.8)	1 (12.5)	
Alpha Defensin (%)			0.41
Unknown	17 (81.0)	8 (100.0)	
Negative	2 (9.5)	0 (0.0)	
Positive	2 (9.5)	0 (0.0)	
ESR mm/h (mean (SD))	36.38 (27.3)	51.50 (31.1)	0.21
CRP (mean (SD))	35.23 (63.0)	36.80 (38.0)	0.95

WBC = white blood cells; PMN = polymorphonuclear cells; ESR = erythrocyte sedimentation rate; CRP = C-reactive protein.

**Table 3:** Initial THA approach and presence of monomicrobial versus polymicrobial cultures.

	THA Anterior Approach	THA Posterior Approach
Monomicrobial	6	4
Polymicrobial	4	4
Total	10	8

**Table 4:** MSIS Outcomes reporting tool utilized for reporting outcomes at 1 year for those patients undergoing surgical intervention after positive *C. acnes* culture.

Tier 1. Infection control with no continued antibiotic therapy
Tier 2. Infection control with the patient on suppressive antibiotic therapy
Tier 3. Need for reoperation and/or revision and/or spacer retention (assigned to subgroups A, B, C, D, E, and F based on the type of reoperation)
(A) Aseptic revision at >1 year from initiation of PJI treatment
(B) Septic revision (including debridement, antibiotics, and implant retention [DAIR]) at >1 year from initiation of PJI treatment (excluding amputation, resection arthroplasty, and arthrodesis)
(C) Aseptic revision at ≤1 year from initiation of PJI treatment
(D) Septic revision (including DAIR) at ≤1 year from initiation of PJI treatment (excluding amputation, resection arthroplasty, and arthrodesis)
(E) Amputation, resection arthroplasty, or arthrodesis
(F) Retained spacer
Tier 4. Death (assigned to subgroups A or B).
Death ≤ 1 year from initiation of PJI treatment
Death > 1 year from initiation of PJI treatment

Legend: Infection management outcome categories, Successful: Tiers 1, 2, Failure directly or indirectly related to PJI or due to secondary causes: Tiers 3A, 3B, 3C, 3D, 3E, 3F, 4A, 4B.

## Data Availability

The original contributions presented in this study are included in the article/Supplementary Material. Further inquiries can be directed to the corresponding author.

## Supplementary Materials

The following supporting information can be downloaded at: https://www.mdpi.com/article/10.3390/antibiotics15020165/s1.

## References

[1] Boddapati V., Fu M.C., Mayman D.J., Su E.P., Sculco P.K., McLawhorn A.S. (2018). Revision Total Knee Arthroplasty for Periprosthetic Joint Infection Is Associated With Increased Postoperative Morbidity and Mortality Relative to Noninfectious Revisions. J. Arthroplast..

[2] Bozic K.J. (2005). The Impact of Infection After Total Hip Arthroplasty on Hospital and Surgeon Resource Utilization. J. Bone Jt. Surg. Am..

[3] Kurtz S.M., Lau E., Watson H., Schmier J.K., Parvizi J. (2012). Economic Burden of Periprosthetic Joint Infection in the United States. J. Arthroplast..

[4] Dodson C.C., Craig E.V., Cordasco F.A., Dines D.M., Dines J.S., DiCarlo E., Brause B.D., Warren R.F. (2010). *Propionibacterium acnes* infection after shoulder arthroplasty: A diagnostic challenge. J. Shoulder Elb. Surg..

[5] Sperling J.W., Kozak T.K., Hanssen A.D., Cofield R.H. (2001). Infection after shoulder arthroplasty. Clin. Orthop..

[6] Aubin G.G., Portillo M.E., Trampuz A., Corvec S. (2014). *Propionibacterium acnes*, an emerging pathogen: From acne to implant-infections, from phylotype to resistance. Méd. Mal. Infect..

[7] Khalil J.G., Gandhi S.D., Park D.K., Fischgrund J.S. (2019). *Cutibacterium acnes* in Spine Pathology: Pathophysiology, Diagnosis, and Management. J. Am. Acad. Orthop. Surg..

[8] Lutz M.-F., Berthelot P., Fresard A., Cazorla C., Carricajo A., Vautrin A.-C., Fessy M.-H., Lucht F. (2005). Arthroplastic and osteosynthetic infections due to *Propionibacterium acnes*: A retrospective study of 52 cases, 1995-2002. Eur. J. Clin. Microbiol. Infect. Dis..

[9] Koh C.K., Marsh J.P., Drinković D., Walker C.G., Poon P.C. (2016). *Propionibacterium acnes* in primary shoulder arthroplasty: Rates of colonization, patient risk factors, and efficacy of perioperative prophylaxis. J. Shoulder Elb. Surg..

[10] Chuang M.J., Jancosko J.J., Mendoza V., Nottage W.M. (2015). The Incidence of *Propionibacterium acnes* in Shoulder Arthroscopy. Arthrosc. J. Arthrosc. Relat. Surg..

[11] Namdari S., Nicholson T., Parvizi J., Ramsey M. (2017). Preoperative doxycycline does not decolonize *Propionibacterium acnes* from the skin of the shoulder: A randomized controlled trial. J. Shoulder Elb. Surg..

[12] Kolakowski L., Lai J.K., Duvall G.T., Jauregui J.J., Dubina A.G., Jones D.L., Williams K.M., Hasan S.A., Henn R.F., Gilotra M.N. (2018). Neer Award 2018: Benzoyl peroxide effectively decreases preoperative *Cutibacterium acnes* shoulder burden: A prospective randomized controlled trial. J. Shoulder Elb. Surg..

[13] Qiu B., Al K., Pena-Diaz A.M., Athwal G.S., Drosdowech D., Faber K.J., Burton J.P., O’Gorman D.B. (2018). *Cutibacterium acnes* and the shoulder microbiome. J. Shoulder Elb. Surg..

[14] Duvall G., Kaveeshwar S., Sood A., Klein A., Williams K., Kolakowski L., Lai J., Enobun B., Hasan S.A., Henn R.F. (2020). Benzoyl peroxide use transiently decreases *Cutibacterium acnes* load on the shoulder. J. Shoulder Elb. Surg..

[15] DiBartola A.C., Swank K.R., Flanigan D.C. (2018). Anterior cruciate ligament reconstruction complicated by *Propionibacterium acnes* infection: Case series. Phys. Sportsmed..

[16] Patzer T., Petersdorf S., Krauspe R., Verde P.E., Henrich B., Hufeland M. (2018). Prevalence of *Propionibacterium acnes* in the glenohumeral compared with the subacromial space in primary shoulder arthroscopies. J. Shoulder Elb. Surg..

[17] Khan U., Torrance E., Townsend R., Davies S., Mackenzie T., Funk L. (2017). Low-grade infections in nonarthroplasty shoulder surgery. J. Shoulder Elb. Surg..

[18] Kajita Y., Iwahori Y., Harada Y., Deie M. (2020). Incidence of *Propionibacterium acnes* in arthroscopic rotator cuff repair. J. Orthop. Sci..

[19] Gausden E.B., Villa J., Warner S.J., Redko M., Pearle A., Miller A., Henry M., Lorich D.G., Helfet D.L., Wellman D.S. (2017). Nonunion After Clavicle Osteosynthesis: High Incidence of *Propionibacterium acnes*. J. Orthop. Trauma.

[20] Elkins J.M., Dennis D.A., Kleeman-Forsthuber L., Yang C.C., Miner T.M., Jennings J.M. (2020). Cutibacterium colonization of the anterior and lateral thigh. Bone Jt. J..

[21] Nodzo S.R., Westrich G.H., Henry M.W., Miller A.O. (2016). Clinical Analysis of *Propionibacterium acnes* Infection After Total Knee Arthroplasty. J. Arthroplast..

[22] Elston M.J., Dupaix J.P., Opanova M.I., Atkinson R.E. (2019). *Cutibacterium acnes* (formerly *Proprionibacterium acnes*) and Shoulder Surgery. Hawaii J. Health Soc. Welf..

[23] Zeller V., Ghorbani A., Strady C., Leonard P., Mamoudy P., Desplaces N. (2007). *Propionibacterium acnes*: An agent of prosthetic joint infection and colonization. J. Infect..

[24] Renz N., Mudrovcic S., Perka C., Trampuz A. (2018). Orthopedic implant-associated infections caused by *Cutibacterium* spp.—A remaining diagnostic challenge. PLoS ONE.

[25] Flurin L., Greenwood-Quaintance K.E., Patel R. (2019). Microbiology of polymicrobial prosthetic joint infection. Diagn. Microbiol. Infect. Dis..

[26] Boisrenoult P. (2018). *Cutibacterium acnes* prosthetic joint infection: Diagnosis and treatment. Orthop. Traumatol. Surg. Res..

[27] Workgroup Convened by the Musculoskeletal Infection Society (2011). New Definition for Periprosthetic Joint Infection. J. Arthroplast..

[28] Batten T.J., Gallacher S., Thomas W.J., Kitson J., Smith C.D. (2022). C.acnes in the joint, is it all just a false positive?. Eur. J. Orthop. Surg. Traumatol..

[29] Portillo M.E., Corvec S., Borens O., Trampuz A. (2013). *Propionibacterium acnes*: An Underestimated Pathogen in Implant-Associated Infections. BioMed Res. Int..

[30] Achermann Y., Liu J., Zbinden R., Zingg P.O., Anagnostopoulos A., Barnard E., Sutter R., Li H., McDowell A., Zinkernagel A.S. (2018). *Propionibacterium avidum*: A Virulent Pathogen Causing Hip Periprosthetic Joint Infection. Clin. Infect. Dis..

[31] Shohat N., Goswami K., Clarkson S., Chisari E., Breckenridge L., Gursay D., Tan T.L., Parvizi J. (2021). Direct Anterior Approach to the Hip Does Not Increase the Risk for Subsequent Periprosthetic Joint Infection. J. Arthroplast..

[32] Namdari S., Nicholson T., Parvizi J. (2020). *Cutibacterium acnes* is Isolated from Air Swabs: Time to Doubt the Value of Traditional Cultures in Shoulder Surgery?. Arch. Bone Jt. Surg..

[33] Liew-Littorin C., Brüggemann H., Davidsson S., Nilsdotter-Augustinsson Å., Hellmark B., Söderquist B. (2019). Clonal diversity of *Cutibacterium acnes* (formerly *Propionibacterium acnes*) in prosthetic joint infections. Anaerobe.

