# Supplementary material for: Cutibacterium acnes Culture Isolation Following Total Hip and Total Knee Arthroplasty
Source: Antibiotics (Basel). 2026 Feb 4;15(2):165. doi: 10.3390/antibiotics15020165 (PMC12937401; doi:10.3390/antibiotics15020165)
Supplement: Supplementary file 1 [file antibiotics-15-00165-s001.zip › antibiotics-4078778-supplementary.pdf]

Supplementary material:

Table S1: Patient baseline, laboratory, and surgical characteristics stratified by MSIS ORT one year outcome.

	**Failure**	**Successful Outcome**	**p-value**
n	6	16	
**Hip vs Knee = Knee (%)**	2 (33.3)	5 (31.2)	0.661
**Sex = Male (%)**	2 (33.3)	6 (37.5)	0.938
**Age (mean (SD))**	65.00 (6.56)	62.59 (11.06)	0.151
**BMI (mean (SD))**	38.95 (7.37)	31.76 (8.93)	0.298
**ASA (mean (SD))**	3.00 (0.00)	2.75 (0.45)	**0.028**
**Polymicrobial (%)**			0.453
No	1 (16.7)	8 (50.0)	
Yes	5 (83.3)	7 (43.8)	
**Prior revision (%)**			0.067
no	2 (33.3)	14 (87.5)	
unknown	1 (16.7)	0 (0.0)	
yes	3 (50.0)	2 (12.5)	
**MSIS criteria for PJI met = yes (%)**	4 (66.7)	10 (62.5)	0.614
**Average time to culture positivity (mean (SD))**	7.21 (3.57)	6.63 (2.35)	0.762
**Synovial fluid cell count (mean (SD))**	25944.33 (33321.40)	25221.75 (40341.64)	0.991
**Percentage PMNs (mean (SD))**	66.83 (39.96)	67.83 (32.11)	0.549
**ESR (mean (SD)**	53.50 (40.68)	32.62 (19.42)	0.246
**CRP (mean (SD))**	72.28 (114.74)	28.08 (28.07)	0.203
**Hip Approach (%)**			0.918
Anterior	2 (50.0)	5 (45.5)	
Posterior	1 (25.0)	5 (45.5)	
Unknown	1 (25.0)	1 (9.1)	

WBC = white blood cells; PMN = polymorphonuclear cells; MSIS = Musculoskeletal Infection Society; ESR = erythrocyte sedimentation rate; CRP = C-reactive protein

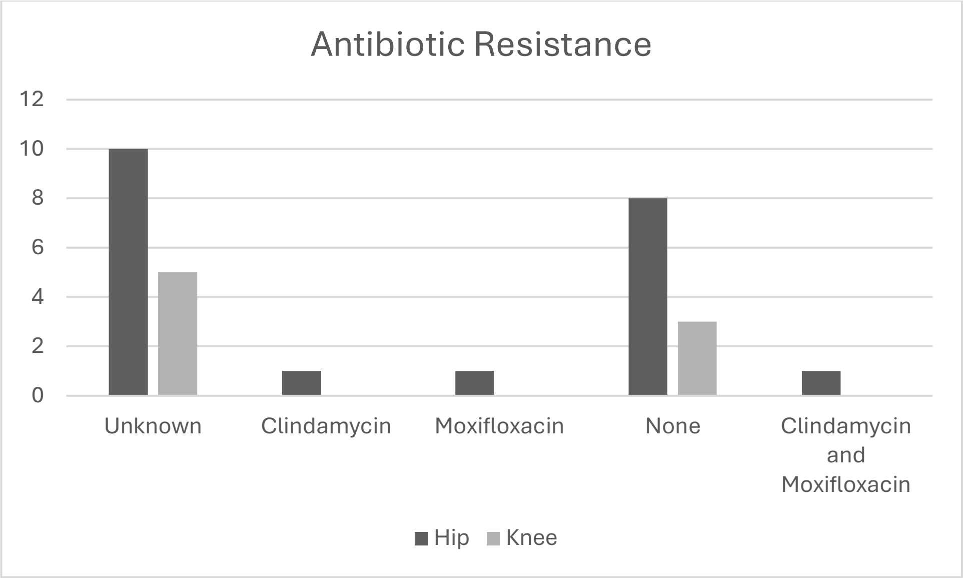

Figure S1 Antibiotic resistance following positive *Cutibacterium* culture
